# Advances in Research on Exosomal miRNAs in Central Nervous System Diseases

**DOI:** 10.1080/17590914.2025.2465546

**Published:** 2025-04-01

**Authors:** Guangli Feng, Xiaoqian Lan, Shiyi Qin, Yuting Shi, Qinxi Zhao, Qing Li, Lianmei Zhong

**Affiliations:** a The First Affiliated Hospital of Kunming Medical University, Kunming, Yunnan, China; b Xuanwu Hospital, Capital Medical University, Beijing, China; c Yunnan Key Laboratory of Stem Cell and Regenerative Medicine, Kunming Medical University, Kunming, Yunnan, China

**Keywords:** Biomarkers, diagnosis and treatment, exosomal miRNAs, neurological diseases

## Abstract

Neurological diseases present a wide range of conditions, intricate diagnosis and treatment processes, and complex prognostic considerations. Therefore, research focusing on the diagnosis and treatment of these diseases is crucial. Exosomal miRNAs are small RNA molecules enclosed in membrane vesicles, released by cells and known to play roles in the development of various neurological disorders. They also serve as specific biomarkers for these conditions. Drawing on extensive research on exosomal miRNAs in diseases like stroke, Alzheimer’s, epilepsy, Parkinson’s, and neuroregeneration, this paper provides a comprehensive review of the relationship between exosomal miRNAs and neurological diseases. We strive to offer current and detailed theoretical understandings to help with the diagnosis and treatment of these disorders.

The blood–brain barrier (BBB) plays a critical role in protecting the brain tissue, but it also limits drug penetration, making it challenging to treat various neurological conditions such as cerebrovascular diseases, gliomas, neurodegenerative diseases, and epilepsy. However, research has shown that exosomal miRNAs have the potential to be used in the diagnosis and treatment of these diseases. Exosomes containing miRNAs can travel through the cerebrospinal fluid, cross the BBB, and be extracted from peripheral blood as biomarkers for brain diseases. These exosomal miRNAs not only serve as non-invasive biomarkers but also play a role in disease treatment by influencing cell growth, differentiation, migration, and apoptosis in the central nervous system (Cui et al., 2021). They have also been identified as potential biomarkers and therapeutic targets for various human diseases, including neurological diseases, gliomas, amyotrophic lateral sclerosis, and cardiovascular diseases (Aili et al., 2021; Chen et al., 2021).

## Biological Characteristics of Exosomal miRNAs

### Biogenesis and Secretion of Exosomes

Extracellular vesicles (EVs) are secreted by various cell types and can be classified into exosomes and microvesicles based on their size and content. Exosomes typically range from 30 to 100 nm in diameter and are primarily involved in RNA transport, whereas microvesicles, which range from 50 nm to 1000 nm, exhibit distinct functional roles compared to exosomes (Van Niel et al., 2018). Exosomes can be discovered in different bodily fluids like blood, urine, saliva, and milk (Ciaccio & Tuttolomondo, 2023). They are generated by various cell types, including neuronal cells, cancer cells, epithelial cells, and chondrocytes. Exosomes have a membrane structure made of phospholipid bilayer and carry a range of bioactive substances, including lipids, messenger RNA (mRNA), microRNA (miRNA), non-coding RNA (ncRNA), and proteins (Théry et al., 2009; Yousif et al., 2021). These substances can be transferred to recipient cells and participate in cellular regulation (Mahjoubin-Tehran et al., 2021). Although initially underestimated, miRNAs regained significant attention in 2007 when Valadi et al. demonstrated that exosomes extracted from mice, when delivered to human mast cells, led to the identification of mouse CDC6 (O89033) and two other distinct murine proteins in human cells (Valadi et al., 2007). This finding provided evidence that exosomal miRNAs can regulate protein translation by modulating mRNA in the cytoplasm. According to the ExoCarta database reported in 2021, various exosomes contain approximately 4946 RNAs, 41860 proteins, 1116 lipids, and various DNA sequences, mRNAs, and miRNAs (Doyle & Wang, 2019). These bioactive exosomes play a crucial role in facilitating cellular communication and material exchange, thereby regulating cellular physiological functions (Wortzel et al., 2019).

Exosomes are formed through a process that begins with the internalization of extracellular materials. The plasma membrane undergoes endocytosis to create early endosomes (EES), which subsequently invaginate and form intralumenal vesicles (ILVs) in the cytoplasm (Mcandrews & Kalluri, 2019). These ILVs are integrated into late endosomes (LES), which are called multivesicular bodies (MVBs). MVBs can either be transported to the trans-Golgi network (TGN) for intracellular cycling, undergo lysosomal degradation or fuse with the plasma membrane to be released into the extracellular space (Yue et al., 2020). The biogenesis and secretion of exosomes require the endosomal sorting complexes required for transport (ESCRT), which are composed of four complexes (ESCRT-0, ESCRT-I, ESCRT-II, and ESCRT-III) and associated proteins (VPS4, Tsg101, and ALIX) (Kalluri & Lebleu, 2020). ESCRT-0 sorts ubiquitinated transport proteins into lipid domains, ESCRT-I and ESCRT-II induce membrane deformation to form stable membrane necks, and the recruitment of the Vps4 complex and ESCRT-III drives membrane neck scission and the dissociation and recycling of ESCRT-III complexes (Juan & Fürthauer, 2018).

The process of exosome formation involves various mechanisms. Some researchers suggest that Rab proteins from GTPase family play a role in mediating the formation of intracellular vesicles (Stenmark, 2009), which then undergo membrane fusion and mature exosome release through protein–protein complexes formed by soluble *N*-ethylmaleimide-sensitive factor attachment protein (SNAP) and its receptors (SNAREs) (Zylbersztejn & Galli, 2011). However, due to the small size of exosomes and the challenges in tracking their budding and secretion, many molecular mechanisms related to exosome formation, secretion, and biological functions are still being investigated.

**Figure 1.**
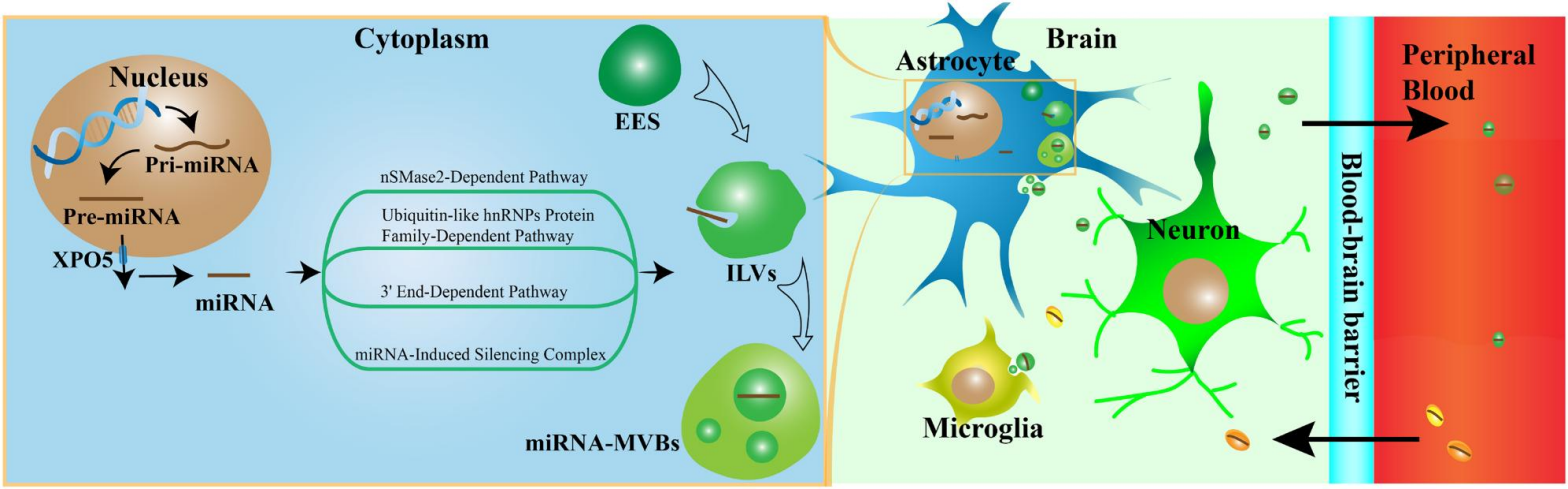
A depiction of how exosomal miRNA is formed and its relationship with the BBB system. The plasma membrane undergoes endocytosis to create early endosomes (EES), which subsequently invaginate and form intralumenal vesicles (ILVs) in the cytoplasm. These intraluminal vesicles (ILVs) are integrated into multivesicular bodies (MVBs). At the same time, the mature miRNA is generated from pri-miRNA. Mature miRNAs can be taken up by firm MVBs through the following four mechanisms. Subsequently, the MVBs combine with the cell membrane and release exosomes containing miRNA. Certain miRNAs have functions in the brain, while others can pass through the blood–brain barrier and appear in the plasma. On the other hand, exosomes derived from different tissues have the ability to cross the blood–brain barrier and target nerve cells.

After exosome generation by cells in the central or peripheral organs, some can cross the blood–brain barrier (BBB) and subsequently appear in plasma or cerebrospinal fluid, exerting distant regulatory effects. Exosomes primarily traverse the BBB via transcytosis, a process in which exosomes bind to receptors on the surface of endothelial cells, enter the cells, and are then released on the opposite side of the barrier through exocytosis (Raposo & Stoorvogel, 2013). However, under pathological conditions such as ischemic stroke or inflammation, the integrity of the blood–brain barrier (BBB) is disrupted, enabling exosomes to traverse through intercellular gaps via the paracellular pathway (Figure 1)(Yang et al., 2019).

### Studies for miRNAs

The first discovered miRNA, lin-4, was identified by the team of Victor Ambros in 1993 in *Caenorhabditis elegans* and characterized as a small RNA approximately 22 nucleotides in length (Lee et al., 1993). In 2001, through high-throughput sequencing, researchers systematically identified miRNAs across multiple species, including humans, fruit flies, and worms, and formally coined the term “miRNA” to refer to these small RNA molecules (Lau et al., 2001). The biogenesis of miRNAs is a complex process involving RNA-modifying enzymes. During transcription by RNA polymerase II (Pol II), a lengthy primary miRNA (pri-miRNA) is synthesized in the nucleus, spanning several hundred nucleotides. This pri-miRNA is then subjected to processing by the RNAase III complex (DRO-SHA) along with cofactors DGCR8, which involves splicing, capping, and polyadenylation, resulting in the formation of a single-stranded precursor miRNA (pre-miRNA) approximately 70–90 nucleotides in length (Denli et al., 2004). Subsequently, the pre-miRNA is exported from the nucleus to the cytoplasm by the exportin protein (XPO5), where the enzyme DICER binds to the end of the pre-miRNA and further processes it into mature miRNA consisting of 20–24 nucleotides (Mahjoubin-Tehran et al., 2021).

These mature miRNAs form RNA-induced silencing complexes (RISCs) with other proteins, which can recognize and bind to the 3′ UTR of target mRNAs, leading to mRNA degradation or inhibition of gene expression (Lee et al., 2004; Rani & Sengar, 2022; Saliminejad et al., 2019). Previous studies have demonstrated that miRNAs play a crucial role in cell cycle regulation; for instance, miR-21 promotes tumor cell proliferation (Iorio & Croce, 2012), while miR-1 and miR-133 modulates muscle cell differentiation (He & Hannon, 2004). Additionally, certain miRNAs regulate apoptosis-related genes, such as miR-34a, which suppresses Bcl-2 and induces cell apoptosis (Bartel, 2004). Moreover, miRNAs have been shown to influence cellular metabolism by modulating the expression of key metabolic enzymes, with miR-122 regulating lipid metabolism in the liver (Bartel, 2004). miRNAs are crucial regulatory molecules in both plants and animals, playing key roles in various physiological and pathological processes. For effective long-range transport and subsequent cellular regulation, the protective bilayer lipid structure of exosomes is essential.

### Exosomal miRNAs

Exosomes have been found to carry proteins, RNAs through proteomic and RNA analyses. When exosomes merge with the extracellular membrane of recipient cells, they transfer their contents including proteins, lipids, nucleic acids, and other molecules influencing gene or protein synthesis and changing cell function. This process facilitates communication between cells (Jeppesen et al., 2019). miRNAs play a vital role in exosome-mediated intercellular communication (Umezu et al., 2013), and their level can vary significantly depending on cell types and pathological conditions. Therefore, modifying miRNAs in exosomes can impact their functions (Gareev et al., 2020; Ouyang et al., 2019). The loading of RNA into exosomes may be facilitated by lipids, depending on lipid vesicles and transport protein domains. Specific nucleotide sequences exhibit stronger affinity for phospholipid bilayers, influenced by factors such as lipid raft structures, hydrophobic modifications, and the physiological concentrations of sphingolipids in raft membranes (Janas et al., 2015). Various mechanisms control the process of packaging miRNAs into exosomes in the cytoplasm, including the following.

#### nSMase2-Dependent Pathway

nSMase2 was the first molecule reported to be associated with miRNA secretion into exosomes. nSMase2 triggers exosome release and mediates miRNA packaging and transfer. Kosaka et al. (2010, 2013) found that overexpression of nSMase2 raises the quantity of exosomal miRNAs, whereas inhibition of nSMase2 expression leads to a decrease in exosomal miRNA levels.

#### Ubiquitin-Like hnRNPs Protein Family-Dependent Pathway

Ubiquitin-like hnRNPA2B1 protein recognizes and binds to the GGAG motif at the 3′ end of miRNA sequences and guides specific miRNAs into exosomes (Villarroya-Beltri et al., 2013). Gao et al. (2018) found that hnRNPA2B1 can identify GUUG sequences in exosomal miRNAs derived from SW620 cells, suggesting its potential involvement in the assembly and release of miRNA. However, the exact mechanism of this pathway remains not entirely understood.

#### 3′ End-Dependent Pathway

miRNAs with specific sequences at their 3′ end (e.g., more poly(U) than poly(A) at the 30′ end) are preferentially sorted into exosomes. Koppers-Lalic et al. discovered that the 3′ ends of uridylated endogenous miRNAs are predominantly present in exosomes derived from B cells or urine, whereas adenylylated endogenous miRNAs’ 3′ ends are more prevalent in B cells. These selection patterns indicate a significant sorting or sequence signal located at the 3′ end of miRNAs, even though the precise mechanism remains unclear (Higa et al., 2014; Koppers-Lalic et al., 2014; Lages et al., 2012).

#### miRNA-Induced Silencing Complex (miRISC)

Mature miRNAs interact with assembly proteins to form miRISC primarily consisting of miRNA, miRNA-silenced mRNA, and AGO2. Guduric-Fuchs found that AGO2 might be involved in sorting and packaging of exosomal miRNAs derived from HEK293T cells (Koppers-Lalic et al., 2014). Further investigations have revealed a close association between miRISC and the sorting and packaging of exosomal miRNA, as Gibbings observed that effective miRISC components exhibits cooperative and co-localized relationships with extracellular vesicles (MVBs). Excrescent MVBs resulting from blocked MVBs’ lysosomal degradation may lead to excessive accumulation of miRISC (Gibbings et al., 2009; Lee et al., 2009). In conclusion, the sorting and packaging of miRNAs into exosomes is heavily influenced by specific sequences within the miRNAs themselves, as well as potential regulation by enzymes or proteins.

## Application of Exosomal miRNAs in Central Nervous System Diseases

### Cerebrovascular Diseases

Cerebrovascular diseases, which are neurological disorders resulting from inadequate blood flow in the brain, are a major contributor to mortality and disability on a global scale. There are essentially two types: ischemic stroke and hemorrhagic stroke. The former one occurs due to occlusion and impaired blood flow leading to hypoxia ischemia brain damage (HIBD), while the latter one results from vascular rupture and subsequent lethal compression of brain tissue. This section focuses on miRNA research related to ischemic stroke.

Nutrition, neuroinflammatory factors, and survival and regrowth of neurons have been identified as key elements that contribute to HIBD based on research findings (Wei et al., 2019), while exosomal miRNAs play a significant role in modulating these elements (Mirzaei et al., 2018). The presence of miR-34c and miR-361 in astrocyte-derived exosomes is essential for supporting neurons and preventing nerve damage following cerebral ischemia (Wu et al., 2020). Another studies by Gamdzyk and Du showed that miR-17-5p from astrocyte-derived exosomes could protect neurons from apoptosis (Gamdzyk et al., 2018) through inhibiting the expression of BNIP-2 (BCL2/adenovirus E1B 19-kDa-interacting protein 2) to protect rat pups neurons from HIBD caused by oxidative stress (Du et al., 2021). Ye et al. discovered that serum exosomal miRNA-27-3p modulate PPARγ expression, exacerbating neuroinflammation in patients with acute cerebral infarction (Ye et al., 2021). Song et al. demonstrated that injecting microglia-derived sEVs into mouse brains right after middle cerebral artery occlusion resulted in a decrease in ischemic brain injury and an enhancement in neuronal survival through exosomal miR-124 and its downstream target USP14 (Song et al., 2019). However, it is important to note that certain sEV-associated factors may actually exacerbate the damage caused by ischemic injury. For example, Xie et al. demonstrated that miR-424-5p carried by exosomes can lead to brain microvascular endothelial cell injury by targeting the FGF2-mediated STAT3 signaling pathway (Xie et al., 2020). In addition to brain-derived exosomal miRNA, there may be extracranial exosomal miRNA that play a role in ischemic stroke. Xin et al. discovered that miRNA-133b present in exosomes derived from bone marrow mesenchymal stem cells (MSCs) could target genes in astrocytes, such as connective tissue growth factor, and genes in neurons, like the RAS gene family A. This targeting led to a decrease in the expression of these genes during ischemic stroke, ultimately reducing the formation of gliotic scars and facilitating the growth of neuronal axons. Such effects are advantageous for nerve repair (Xin et al., 2013). Likewise, exosomes isolated from MSCs loaded with miR-146a-5p have been shown to decrease neuronal apoptosis and hinder inflammation associated with microglial M1 polarization by reducing IRAK1 and NFAT5 expression, thus providing neuroprotection and promoting functional recovery (Duan et al., 2020). Understanding these regulatory functions is crucial as they can help determine gene therapy approaches in the future.

Despite having a regulatory function, the focus of much research has been on the expression level of miRNAs and their potential as medical biomarkers. In an observational study by Zhou et al., it was discovered that exosomal miR-134 significantly increases 24 hours after stroke onset in acute ischemic stroke (AIS) patients compared to control. Furthermore, elevated levels of miR-134 in stroke patients were linked to a worse prognosis (Zhou et al., 2018). The retrospective case–control study by Chen et al. revealed a correlation between heightened levels of exosomal miR223 and acute ischemic stroke (Chen et al., 2017). The exosomes of patients with ischemic stroke were found to contain high levels of hsa-miR-15b-5p, hsa-miR-184, and hsa-miR-16-5p, according to the research conducted by Jiang et al. (2022). Ji et al. found that the serum levels of exosomal miR-9 and miR-124 were significantly higher in patients with ischemic stroke than in controls, whose levels were also correlated with NIHSS scores and the volume of the infarct (Ji et al., 2016). Rather than being upregulated, certain miRNA demonstrate downregulation. For example, Wang et al. uncovered the significance of serum exosomal miR-328-3p in predicting short-term outcomes for individuals with stroke (Wang et al., 2021). In order to gain a deeper understanding of the nuanced roles of miRNA, researchers have conducted studies to examine the different subtypes of miRNA and how their expressions change at various stages of diseases (refer to Ciaccio and Tuttolomondo (2023) for more information).

Up to this point, there has been a lack of research on miRNA and hemorrhagic stroke. One research showed that activated microglia could secrete exosomal miR-383-3p to aggravate neuronal necrosis by inhibiting ATF4 expression (Wei et al., 2021). In rats suffered from brain hemorrhage, enhancing expression of miR-146a may effectively inhibit inflammation and oxidative stress caused by brain hemorrhage, thereby protecting brain tissue (Duan et al., 2020). One latest research demonstrates that exosomal miR-27a had a therapeutic effect on stroke recovery by promoting axonal remodeling and improving neurological outcomes (Zhang et al., 2025). Further efforts are required to better elucidate the role of miRNA in hemorrhagic stroke.

### Alzheimer’s Disease

AD, a neurodegenerative condition that usually manifests in older individuals, is primarily characterized by the formation of senile plaques (SPs), neurofibrillary tangles (NFTs), impaired synaptic function, neuronal degeneration, ongoing inflammation, and reduction in brain volume. SPs consist of amyloid beta (Aβ) oligomers, which disrupt neuronal communication between synapses, leading to synaptic dysfunction and neuronal death (Xu et al., 2021; Van Der Kant et al., 2020). The primary pathological characteristics involve the aggregation of Aβ and the phosphorylation of tau protein, leading to neuronal apoptosis (Jiang et al., 2019). Research has shown that miRNAs released from exosomes regulate the expression and function of amyloid precursor protein (APP) and tau protein (Chen et al., 2019). In order to improve the clinical diagnosis of AD, numerous research studies have concentrated on biomarkers derived from neuroimaging, cerebrospinal fluid, and peripheral blood. With their advantages in disease diagnosis, miRNAs are gaining more attention for detection. Exosome-derived miRNAs have become essential in understanding and treating the pathogenesis of AD. Exosomes, serving as nanoscale carriers of miRNAs, offer potential avenues for researching the mechanisms, diagnosis, and treatment of AD (Table 1).

**Table 1.** miRNAs involved in AD diagnosis.

Molecule	Subject	Level	Function	References
miR-342-3p	AD patient	Down-regulated	Biomarkers for AD diagnosis	Riancho et al. (2017)
miR-9-5p and miR-598	AD patient	Up-regulated	Biomarkers for AD diagnosis	Riancho et al. (2017)
miR-455-3p	AD patients	Up-regulated	Biomarkers for AD diagnosis	Kumar and Reddy (2021)
miR-135a and miR-384	AD patients	Up-regulated	Distinguishing AD	Yang et al. (2018)
miR-193b	AD patients	Down-regulated	Biomarkers for AD diagnosis	Yang et al. (2018)
miR-135a	AD patients	Up-regulated	AD screening	Liu et al. (2021)
miR-212 and miR-132	AD patients	Down-regulated	Biomarkers for AD diagnosis	Cha et al. (2019)
MiR-30b-5p	AD patients	Down-regulated	Biomarkers for AD diagnosis	Dong et al. (2021)
miR-125b-5p, miR-501-3p, and miR-455-5p	AD patients	Up-regulated	Biomarkers for AD diagnosis	Takousis et al. (2019)
miR-138-5p, miR-181c-5p, and miR-29c-3p	AD patients	Down-regulated	Biomarkers for AD diagnosis	Takousis et al. (2019)
miR-16-5p	AD patients	Only down-regulated in early-stage	Early diagnosis for AD	McKeever et al. (2018)
miR-193b	AD patients	Down-regulated	Non-invasive biomarker for MCI and DAT diagnosis	Liu et al. (2014)
miR-328-3p, miR-320a, and miR-204-5p	AD patients	Down-regulated	AD and FTD diagnosis	Tan et al. (2021)

miRNAs may act as biomarkers for diagnosing AD. Riancho et al. analyzed exosome miRNAs in cerebrospinal fluid (CSF) from individuals with Alzheimer’s disease (AD), discovering significant decreases in miR-342-3p and increases in miR-9-5p and miR-598 in comparison to the controls (Riancho et al., 2017). The research conducted by Kumar et al. revealed higher levels of miR-455-3p in the serum and CSF of Alzheimer’s disease patients, indicating that miR-455-3p could potentially be used as a diagnostic biomarker for AD (Kumar & Reddy, 2021). Yang et al. (2018) observed upregulation of miR-135a and miR-384 and downregulation of miR-193b in serum exosomes of AD patients, with miR-384 being the best biomarker among the three miRs for distinguishing AD. Liu’s research indicated that miR-135a expression is significantly higher in AD serum, proposing it as a potential serum biomarker for AD screening (Liu et al., 2021). Cha et al. found that miR-212 and miR-132 are both downregulated in exosomes from AD brain tissue, with miR-212 showing significant dysregulation (Cha et al., 2019). miR-30b-5p displays varying expression levels in neurodegenerative diseases such as Alzheimer’s disease, Parkinson’s disease, multiple sclerosis, and amyotrophic lateral sclerosis (ALS). It is notably decreased in the serum or plasma of patients with Alzheimer’s disease, Parkinson’s disease, and ALS (Dong et al., 2021). Takousis et al. performed a meta-analysis revealing significant upregulation of miR-125b-5p, miR-501-3p, and miR-455-5p, and significant downregulation of miR-138-5p, miR-181c-5p, and miR-29c-3p in AD brain tissue. Among these molecules, miR-125b-5p, which is highly abundant in the brain, is mainly found in neurons, astrocytes, and microglia (Wang et al., 2020). Besides, in cerebrospinal fluid, miR-598-3p, miR-451a, and miR-127-3p showed significant downregulation, whereas miR-342-3p and miR-107 exhibited significant downregulation in the bloodstream (Takousis et al., 2019). Mckeever et al. noticed that miR-16-5p was only downregulated in early-stage patients but returned to normal levels in late-stage patients, indicating it may need further study as an early marker of AD (McKeever et al., 2018). Lui et al. found that miR-193b is downregulated in serum exosomes of patients with mild cognitive impairment (MCI) and Alzheimer’s type dementia (DAT). Compared to MCI patients, those with DAT consistently exhibit lower cerebrospinal fluid levels of miR-193b, suggesting that it may serve as a non-invasive biomarker for differentiating MCI from DAT (Liu et al., 2014). Tan found that in AD and frontotemporal dementia (FTD) patients, CSF-derived exosomal miRNAs such as miR-328-3p, miR-320a, and miR-204-5p were significantly decreased compared to controls, and miR-320a was significantly decreased in FTD patients. These miRNAs may serve as potential differential diagnosis biomarkers for AD and FTD (Table 2) (Tan et al., 2021).

**Table 2.** miRNAs participated in AD process.

Molecule	Source	Subject	Function	References
miR-223	Mesenchymal stem cells	AD patient	Protects neurons from apoptosis	Wei et al. (2020)
miR-29b	Brain tissues	AD rat models	Has a protective effect on cognitive function	Jahangard et al. (2020)
miR-125b-5p	Cerebrospinal fluid	AD patients	Mainly exacerbates Tau protein phosphorylation and oxidative stress	Wang et al. (2020), Shen et al. (2018)
miR-146a	Choroid plexus cells	AD mouse models	Alleviates astrocyte inflammation and induces increased Aβ tolerance	Nakano et al. (2020), Kubota et al. (2018) Yang et al. (2021)
miRNA-22	Adipose-derived mesenchymal stem cells	AD mouse models	Improves neurofunction and neuroinflammation	Zhai et al. (2021)
miR-29b-2	Plasma and cerebrospinal fluid	AD patients, 3xTg-AD mouse model	Inhibits of beta-amyloid oligomers production	Kiko et al. (2014) Lin et al. (2024)
miR-34a	Cerebrospinal fluid and plasma	AD mouse models	Promotes the amyloidogenic processing of APP	Jian et al. (2017) Xu et al. (2018)
miR-124-3p	Mixed tissue of bilateral cerebral cortex and hippocampus	An rmTBI mouse model	Mitigates β-amyloid abnormalities	Ge et al. (2020)

miR-139 is upregulated in AD and targets cannabinoid receptor 2 (CB2R), impairing learning and memory formation in the hippocampus (Tang et al., 2017). Liu et al. found that miR-455-3p was significantly upregulated in exosomes from cerebrospinal fluid and serum of AD patients and AD mouse models. Through the high-dose injection of exosomal miR-455-3p into the lateral ventricles, it has been further demonstrated that exosome carriers are capable of crossing the blood–brain barrier. This discovery provides a solid foundation for the advancement of exosome-based drug delivery systems (Liu et al., 2021). Reports suggest that microglial exosomes play a role in Alzheimer’s disease. In vitro studies show that inflammatory mediators produced by amyloid precursor protein (APP) and β-amyloid protein (Aβ) induce microglial activation and secretion of miR-21 exosomes, affecting AD progression (Fernandes et al., 2018). During AD pathogenesis, miR-34a promotes the amyloidogenic processing of APP, and knockdown of miR-34a can reduce APP accumulation in brain tissue (Jian et al., 2017; Xu et al., 2018). A study demonstrated that miR-124-3p in microglial exosomes targets Rela, an inhibitory transcription factor of ApoE that facilitates β-amyloid proteolytic breakdown, thereby mitigating β-amyloid abnormalities in Alzheimer’s disease associated with repetitive mild traumatic brain injury (rmTBI) (Ge et al., 2020).

Furthermore, specific exosomal miRNA have been identified to be involved in particular processes in AD, suggesting they could serve as potential therapeutic targets. Exosomal miR-223 derived from mesenchymal stem cells (MSCs) protected neuronal cells from apoptosis through the PTEN-PI3K/Akt pathway (Wei et al., 2020). Jahangard’s research found that miR-29b is downregulated in AD patients and that bilateral injection of exosome miR-29b into the brain of AD rat models has a protective effect on cognitive function (Jahangard et al., 2020). Besides, Takehiro et al. firstly discovered that miR-29a and miR-29b may facilitate increases in both amyloid (Kiko et al., 2014). By engineering exosomes containing miR-29b-2 and applying them in the 3xTg-AD animal model, Lin et al. recently discovered a decrease in the translational level of PSEN1 and subsequent inhibition of beta-amyloid oligomers production (Lin et al., 2024). Overexpression of miR-125b-5p in AD patients’ CSF mainly exacerbates Tau protein phosphorylation, neuronal apoptosis, and induces memory loss and cognitive impairment (Wang et al., 2020). Another study suggests that inhibiting miR-125b-5p can reduce ROS levels and mitochondrial membrane potential, thereby providing neuroprotection against oxidative stress (Shen et al., 2018). Lugli et al. found that overexpression of miR-125b-5p can lead to memory deficits in mice, indicating that exosome-mediated miR-125b-5p may be involved in the onset and progression of AD (Lugli et al., 2015). miR-125b-5p could be a novel regulatory factor in AD progression and a potential therapeutic target for AD treatment. Moreover, miR-146a acts as an anti-inflammatory miRNA, and it can be secreted by choroid plexus (CP) cells into the CSF. This exosomal miR-146a helps regulate astrocyte inflammation through NF-κB, enhances synaptic growth, and alleviates cognitive impairment. Noticeably, upregulation of serum exosomal miR-146a levels and improvement of astrocyte damage can prevent diabetes-induced cognitive dysfunction (Nakano et al., 2020; Kubota et al., 2018). However, additional research revealed that the upregulation of miR-146a was a result of the release of bone marrow mesenchymal stem cells (BMSCs), which improved cognitive deficits in AD mouse models by boosting miR-146a expression in the hippocampus (Nakano et al., 2020). Exosomal miR-223 derived from mesenchymal stem cells (MSCs) protected neuronal cells from apoptosis through the PTEN-PI3K/Akt pathway (Wei et al., 2020). Nervetheless, Yang et al. found that circulating exosomal miR-146a induces increased Aβ tolerance in inflammatory AD, resulting in impaired Aβ clearance and promoting AD progression (Yang et al., 2021). Zhai et al. discovered that exosomal miRNA-22 derived from adipose-derived mesenchymal stem cells (ADMSCs) effectively improves neurofunction and neuroinflammation in AD mouse models (Zhai et al., 2021).

### Epilepsy

Epilepsy is a clinical syndrome that is defined by the highly synchronized abnormal discharge of brain neurons, which can be caused by a variety of factors. The mechanisms underlying epilepsy are intricate and can include damage to the blood–brain barrier, inflammatory responses in the central nervous system, demyelination, and injury to neurons (Table 3) (Rana & Musto, 2018; Kanner, 2012).

**Table 3.** miRNAs involved in AD diagnosis.

Molecule	Source	Level	Subject	Function	References
miR-21-5p, miR-146a-5p	Cerebrospinal fluid	Up-regulated	Epilepsy patients	Biomarkers for epilepsy diagnosis	Raoof et al. (2017)
miR-21-5p, miR-146a-5p	Serum	Up-regulated	Epilepsy patients	Biomarkers for epilepsy diagnosis	Raoof et al. (2017)
miR-132	Hippocampus	Up-regulated	Epilepsy patients and a rat TLE model	Biomarkers for epilepsy diagnosis	Korotkov et al. (2020)
miR-106b-5p	Serum	Up-regulated	Epilepsy patients	Biomarkers for epilepsy diagnosis	Wang et al. (2015)
miR-15a-5p	Serum	Down-regulated	Epilepsy patients	Biomarkers for epilepsy diagnosis	Wang et al. (2015)
miR-129-5p, miR-214-3p, miR-219a-5p, and miR-34c-5p,	Plasma	Up-regulated	Epilepsy patients	Biomarkers for epilepsy diagnosis	Huang et al. (2020)
miR-421, miR-184	Plasma	Down-regulated	Epilepsy patients	Biomarkers for epilepsy diagnosis	Huang et al. (2020)
miR-132	Culture medium	Up-regulated	Cell model of status epilepticus	Increases frequency of epileptic discharges	Xiang et al. (2015)
miRNA-134	Hippocampus	Up-regulated	Epilepsy patients	The silencing of miR-134 demonstrated anticonvulsant and neuroprotective effects	Jimenez-Mateos et al. (2012)
miR-142-3p, miR-223-3p, and miR-21-5p	TSC tubers	Up-regulated	TSC patients	Promote the neuroinflammatory cascades of epilepsy	Cukovic et al. (2021)
miR-346, miR-331-3p.	Forebrain	Down-regulated	A rat model of chronic temporal lobe epilepsy	Influence epilepsy development	Gitaí et al. (2020)
hsa-miR-184	Hippocampus	Down-regulated	Patients with mTLE	Suppress cytokine release from primary microglia	Danis et al. (2016)
miR-204, miR-218	Hippocampus	Down-regulated	Patients with mTLE + HS	Regulate axonal guidance and synaptic plasticity	Kaalund et al. (2014)

Currently, there is ongoing research regarding the alterations in miRNA levels following epilepsy. Significant differences in exosomal miRNA expression have been observed in the blood of temporal lobe epilepsy patients and in the cerebrospinal fluid (CSF) of individuals with status epilepticus, according to research findings (Yan et al., 2017). Interestingly, epilepsy patients exhibit elevated expression levels of miR-21-5p and miR-146a-5p in their cerebrospinal fluid, while showing increased levels of miR-146a-5p and miR-194-5p in their serum (Raoof et al., 2017). Korotkov found significant upregulation of miR-132 in epilepsy (Korotkov et al., 2020). Wang et al. reported that in different epilepsy subtypes, miR-106b-5p is upregulated while miR-15a-5p is downregulated in the serum, with miR-106b-5p showing significant sensitivity (80%) and specificity (81%) (Wang et al., 2015). Huang et al. analyzed exosomal miRNAs from the plasma of patients with mesial temporal lobe epilepsy with hippocampal sclerosis (mTLE + HS) and found upregulation of miR-129-5p, miR-214-3p, miR-219a-5p, and miR-34c-5p, and downregulation of miR-421 and miR-184. Among these, miR-184 has better specificity and diagnostic value (Huang et al., 2020). These six miRNAs may target related signaling pathways such as hippo, p53, TGF-β, HIF-1, and mTOR to regulate the progression of mTLE + HS (Huang et al., 2020).

Martins-Ferreira et al. found that miR-146a, miR-155, and miR-132 might be involved in the occurrence of genetic generalized epilepsy (GGE) and reported that these three circulating miRNAs have potential value as biomarkers for GGE (Martins-Ferreira et al., 2020). Following this, Xiang et al. discovered that when miR-132 was overexpressed in a hippocampal neuronal culture model of status epilepticus, there was a significant increase in the frequency of epileptic discharges, resembling the post-epileptic enhancement of high-voltage-activated Ca^2+^ current (Xiang et al., 2015). These findings suggest that miR-132 may play a role in the development of epilepsy.

Moreover, Jimenez-Mateos observed an upregulation of miRNA-134 in experimental epilepsy and resected hippocampi from epilepsy patients. The silencing of miR-134 demonstrated anticonvulsant and neuroprotective effects (Jimenez-Mateos et al., 2012). Cukovic et al. discovered that miR-142-3p, miR-223-3p, and miR-21-5p were notably elevated in epileptogenic tuberous sclerosis complex (TSC) nodules compared to non-epileptogenic ones, suggesting their role in promoting the neuroinflammatory cascades of epilepsy (Cukovic et al., 2021). Gitaí et al. examined exosomal miRNAs from prefrontal cells in a rat model of chronic temporal lobe epilepsy and observed a significant decrease in miR-346 and miR-331-3p. These miRNAs could potentially influence epilepsy development by modulating signaling pathways linked to GABA (targeted by miR-346) and mTOR (targeted by miR-331-3p) (Gitaí et al., 2020). Danis discovered that hsa-miR-184 expression was notably reduced in both mTLE + HS and mTLE-HS. Additionally, the overexpression of hsa-miR-184 successfully suppressed cytokine release from primary microglia without impacting neuronal and astrocyte function (Danis et al., 2016). Furthermore, in Kaalund’s study, it was discovered that there was a notable decrease in the expression of miR-204 and miR-218 in the hippocampal tissues of patients with mTLE + HS. It is suggested that miR-218 may play a role in regulating axonal guidance and synaptic plasticity by targeting genes such as ROBO1, GRM1, SLC1A2, and GNAI2 (Kaalund et al., 2014).

More extensive research is required on exosomal miRNA and epilepsy, as current investigations are limited. Large-scale studies should be conducted to explore various factors such as different models, brain regions, sample sizes, and lifestyle, in order to gain a more comprehensive understanding of epilepsy at the genetic level.

### Parkinson’s Disease

Parkinson’s disease (PD) is the second most prevalent neurodegenerative condition following Alzheimer’s disease (AD), with a prevalence of approximately 1–2% among individuals aged 60 and above globally (Angelopoulou et al., 2019). Presently, the focus of Parkinson’s disease treatments is on easing symptoms, particularly those related to motor function, as there is currently no cure for the disease. Hence, it is crucial to create new medications that can alter the advancement of the disease. Due to their capacity to traverse the blood–brain barrier, exosomes containing miRNA are being considered as a promising option for drug delivery systems and gene therapy.

Throughout the investigation into exosomal miRNAs and PD, Gui et al. were the pioneers in identifying a notable reduction in the levels of miR-1 and miR-19b-3p in exosomes originating from the cerebrospinal fluid (CSF) of PD individuals. Conversely, miR-153, miR-409-3p, miR-10a-5p, and let-7g-3p were markedly elevated in exosomes derived from the CSF of PD patients (Gui et al., 2015). miR-124, a highly abundant miRNA in the brain, plays a role in various processes such as neurogenesis, synaptic morphology, neurotransmission, inflammation, autophagy, and mitochondrial function. Clinical evidence indicates that lower plasma levels of miR-124 may be used as a potential diagnostic biomarker for PD (Dorsey et al., 2018). Moreover, there are other miRNA that display significant differences in expression levels between individuals diagnosed with PD and those who do not have the disease. One finding has revealed a decrease in miR-133 expression specifically in the brain tissue of patients with Parkinson’s disease as opposed to control groups (Kim et al., 2007). Ma et al. discovered that PD patients had significantly lower serum levels of miR-221-3p compared to healthy controls, indicating that the downregulation of miR-221-3p could serve as a potential biomarker for assessing PD (Ma et al., 2016). Xie et al. observed a reduction in miR-15b-5p levels in the serum exosomes of PD patients when compared to healthy controls, indicating that miR-15b-5p may hold promise for both diagnosing and treating PD (Xie et al., 2022). Shu et al. found that in early-stage PD patients, serum levels of miR-132-3p and miR-146a-5p were significantly lower than those in healthy individuals. These levels were further reduced in severe PD patients compared to early-stage patients. Additionally, miR-132-3p and miR-146a-5p expression negatively correlated with Braak stages, suggesting these two miRNAs might be potential biomarkers for PD detection (Shu et al., 2020). Ozdilek found that miR-29c was significantly upregulated in the serum of Turkish PD patients (Ozdilek & Demircan, 2021), though other studies reported downregulation of miR-29c (Bai et al., 2017; Botta-Orfila et al., 2014), indicating variability in miRNA expression across different studies, possibly related to different populations or disease stages.

Some research also investigates the role of miRNAs in distinguishing between PD and other diseases. Marques et al. analyzed miRNA sequencing data from cerebrospinal fluid samples of patients with PD and multiple system atrophy (MSA). They discovered that miR-24 was notably decreased in PD, whereas miR-19a, miR-19b, miR-24, and miR-34c were all significantly reduced in MSA. Additionally, they highlighted miR-24 and miR-148 as potentially linked to cerebellar ataxia (Marques et al., 2017). Because of the similarity in clinical symptoms between PD and progressive supranuclear palsy (PSP), a more effective method of differentiation is needed for these two diseases. In their analysis of exosomal miRNAs from peripheral blood samples, Manna et al. (2021) discovered that miR-22-3p was notably elevated in both PD and PSP patients when compared to the healthy control group. On the other hand, miR-21-3p levels were significantly decreased in PD but increased in PSP. These findings indicate that miR-21-3p and miR-22-3p may serve as useful markers for distinguishing between PD and PSP (Table 4) .

**Table 4.** miRNAs participated in PD process.

Molecule	Subject	Mechanism	References
miR-23b-3p	Rat model of PD	Inhibit α-syn expression	Cai et al. (2021)
miR-7	Rat model of PD, PD patient	Downregulate α-syn formation	McMillan et al. (2017)
miR-16-1	PD patient	Increase α-syn protein level	Vilaça-Faria et al. (2019)
miR-137	Mouse model of PD	Aggravate oxidative stress damage	Jiang et al. (2019)
miR-124-3p	Cells model of PD	Protect neuron from death	Dong et al. (2018)
miR-221-3p	Cells model of PD	Inhibit neuroinflammation via mediating microglia	Lang et al. (2020), Li et al. (2018)
miR-188-3p	Cells model of PD	Inhibit cell autophagy and inflammasome activity	Li et al. (2021)
miR-218-5p	Cat model of PD	Alleviate damages to dopaminergic neurons	Ma et al. (2021)
miR-212-5p	Mouse model of PD	Protect dopaminergic neuron	Sun et al. (2018)
miR-200a-3p	Cells model of PD	Decrease neuronal apoptosis	Shakespear et al. (2020)

As more truths are uncovered, the roles of exosomal miRNA in the progression of PD are becoming increasingly clear. By targeting and inhibiting α-syn expression, miR-23b-3p in neural stem cells has been demonstrated to provide neuroprotective effects (Cai et al., 2021). Mcmillan et al. discovered that exosomal miR-7 could potentially reduce α-synuclein formation in a rat model of Parkinson’s disease. This was further supported by the observation of decreased miR-7 expression in PD patients (McMillan et al., 2017). Likewise, increased levels of miR-16-1 block the translation of HSP70 mRNA (which inhibits α-syn), resulting in higher α-syn protein levels and advancing PD development (Vilaça-Faria et al., 2019). Exosomal miRNAs were observed to adjust to variations in the inflammatory microenvironment. Jiang et al. found that exosomal miR-137 is able to target and suppress oxidative stress response factor 1 (OXR1), and the decrease in exosomal miR-137 levels helps mitigate oxidative stress damage in PD through the upregulation of OXR1 (Jiang et al., 2019). The neuroprotective effects of miR-124-3p are significant in PD models, as they affect the death of dopaminergic cells, dysregulation of autophagy, neuroinflammation, and oxidative damage (Dong et al., 2018). A study carried out in mouse models and human cell lines found that miR-221-3p levels are decreased in Parkinson’s disease models. This microRNA shows neuroprotective properties by inhibiting neuroinflammation through the modulation of microglia (Lang et al., 2020). Moreover, Li et al. observed that miR-221 plays a role in regulating PC12 cell viability and apoptosis in a 6-OHDA-treated PD cell model by targeting PTEN. This suggests that targeting miR-221 could be a potential therapeutic strategy for PD (Li et al., 2018). Additionally, Li’s research on MPTP-induced PD mouse models and MPP+-induced PD MN9D cell models utilizing miR-188-3p-modified adipose-derived MSC exosomes revealed that miR-188-3p could potentially suppress cell autophagy and inflammasome activity by directly targeting NLRP3 and CDK5, offering a protective effect against PD (Li et al., 2021). Dopaminergic hypofunction is the central feature of PD, and certain miRNA have been shown to regulate this dysfunction. In their study, Ma and Zhang determined that the inhibition of LASP1 by miR-218-5p could mitigate injuries to dopaminergic neurons in rats with Parkinson’s disease (Ma et al., 2021). Sun et al. (2018) demonstrated that in Parkinson’s disease models, miR-212-5p has the potential to suppress the expression and function of SIRT2. This action helps safeguard dopaminergic neurons from degeneration, leading to a decrease in DAT levels by modulating the SIRT2/p53 pathway. In his study, Shakespeare found that astrocyte-derived exosomal miR-200a-3p inhibited MKK4 expression by binding to two distinct sites on the Map2k4/MKK4 mRNA 3′-UTR, leading to a decrease in neuronal apoptosis induced by MPP+ in a cellular model of PD. This highlights the neuroprotective effects of the exosomal miR-200a-3p (Shakespear et al., 2020).

Recently, several research studies have been carried out to explore the potential application of exosomal miRNA in treating PD. According to Xie et al., miR-15b-5p, miR-30c-2-3p, miR-138-5p, and miR-338-3p have been predicted to potentially function as biomarkers for Parkinson’s disease. These microRNAs may play a role in regulating disease progression through their interactions with target genes such as GNAI3, ADCY5, UBE2J1, PRKCA, PPP2R1B, GNG2, CREB3L2, and GNG12 (Xie et al., 2022). According to Yao’s findings, miR-124 has the potential to be a valuable therapeutic target for modulating neuroinflammatory responses in Parkinson’s disease by targeting p62, p38, and autophagy pathways (Yao et al., 2019).

### Current Challenges

Exosomal miRNAs have garnered significant attention in therapeutic and diagnostic research due to their pivotal role in intercellular communication and gene expression regulation. However, their clinical translation faces several challenges. Firstly, in terms of extraction, exosome concentrations in body fluids such as blood and urine are low, necessitating efficient enrichment methods (Li et al., 2017). Moreover, the heterogeneity of exosomal miRNA content (even within the same cell type, exosomes can secrete different types and quantities of miRNAs) complicates the process (Kowal et al., 2016). Current methods, including ultracentrifugation and commercial kits, are costly and complex. Secondly, despite the dual lipid-layer protection of the exosomal membrane, exosomal miRNAs remain susceptible to inactivation due to pH changes or enzymatic degradation, which limits their transcriptional efficacy (O’Brien et al., 2020). Furthermore, there is a lack of effective targeting strategies to prevent uptake by non-target tissues (He et al., 2022). Therefore, research on enhancing the targeting efficiency of exosomal miRNAs is essential. For example, Yang et al. (2017) demonstrated that modified exosomes, with rabies virus glycoprotein (RVG) fused to the exosomal protein lysosome-associated membrane glycoprotein 2b (Lamp2b), could efficiently deliver miR-124 to the infarct site, acquiring neuronal identity and protecting against ischemic injury. However, it is important to note that physical or chemical modifications to the exosomal membrane may compromise its structural integrity, reducing modification efficiency and posing challenges for large-scale production (Johnsen et al., 2014). Addressing these challenges requires the development of more efficient, cost-effective, and stable methods for extraction, modification, and delivery, alongside further research into the in vivo behavior of exosomal miRNAs, to facilitate their clinical application.

## Summary and Prospects

Ever since the idea of gene therapy was first introduced in 1972, there has been notable advancement in its use as an innovative treatment method. However, the success of gene therapy largely depends on the efficiency of the vectors used for gene transfer (Yu et al., 2021). Compared to viruses, bacteria, phages, and synthetic lipids, exosome-based delivery systems stand out as safer, more specific, and more efficient gene delivery carriers. Not only do they exhibit stable and biocompatible cell-to-cell communication functions, but they also have the capability to traverse biological barriers and target particular cells and organs. Moreover, the membrane of exosomes can be manipulated to express or absorb specific molecules, offering a promising approach for developing drug-loaded exosome technologies.

As more research delves into exosome-mediated neural pathways, it has been discovered that blood, urine, and cerebrospinal fluid contain high levels of exosomal miRNAs. Some of these miRNAs can be used as biomarkers for early diagnosis and for assessing prognosis in the clinical management of central system diseases. Given the strictly regulated spatio-temporal expression patterns of miRNAs, there remains considerable potential for further research to examine changes in miRNA expression levels during different stages of central nervous system diseases and the related regulatory pathways.

Furthermore, exosomes possess the potential to penetrate the blood–brain barrier and their low immunogenicity make them a promising option for delivering drugs to address neurodegenerative disorders such as Alzheimer’s disease, Parkinson’s disease, and epilepsy. As interdisciplinary fields continue to progress, some researchers have been working on developing cell and exosome implantation techniques for functional repair following nerve damage, yet the clinical results have been less than optimal. Nevertheless, the advancement of biomimetic scaffold materials featuring three-dimensional structures is on the rise. In rodent models, these scaffolds have been shown to enhance axon regeneration and restore limb motor function after experimental spinal cord or sciatic nerve injury. These materials can be able to serve as a matrix for cell growth, store factors necessary for cell proliferation and differentiation, and load specific exosome miRNAs for the purpose of repair and neural regeneration. This is emerging as a key area of interest for upcoming research projects. miR-22-3p, for example, promotes and stabilizes neuronal regeneration (Li et al., 2020). Some researchers have found that MSC-derived exosome miR-17-92 can inhibit PTEN gene expression in neurons, further promoting axon growth and accelerating nerve repair (Bucan et al., 2019). This finding provides a potential application for exosome miRNAs in neural damage repair. Our research team has made significant advancements in peripheral nerve repair and is currently analyzing exosomal miRNAs from clinical samples using high-throughput sequencing to pinpoint key miRNAs, create targeted carriers for exosomes, and study central nervous system disorders.

In conclusion, extensive research demonstrates the involvement of exosomes and exosomal miRNAs in the progression of neurological diseases, emphasizing their essential roles in the development enhancement of neuroclinical medicine and neuropathology. Utilizing exosomes for transporting biomarkers for diagnostic purposes, and exploring their potential as vehicles for delivering drug or gene therapy to targeted brain regions or cell types in neurological treatments, shows great potential. Despite the clear advantages of exosomal miRNA, the intrinsic limitations from exosomes must also be considered. Consequently, despite the great potential of exosomes as essential carriers in gene therapy, the use of exosomal miRNAs for the treatment of certain diseases still poses challenges that demand further investigation and breakthroughs in exosome purification, low loading efficiency, poor targeting, and exosome heterogeneity by researchers and clinicians.

